# A Case of Malignant Diphtheria Saved by Antitoxin, Indicating the Great Value of the New Remedy

**Published:** 1895-03

**Authors:** Samuel G. Dorr

**Affiliations:** Buffalo, N. Y.


### A CASE OF MALIGNANT DIPHTHERIA SAVED BY ANTITOXIN, INDICATING THE GREAT VALUE OF THE NEW REMEDY.

By SAMUEL G. DORR, M. D., Buffalo, N. Y.

I WAS called January 16, 1895, to see a thirteen year old girl living at 426 William street. At that time there was a slight swelling of the tonsils, with a small amount of redness, but no grave symptoms. I prescribed the ordinary treatment for such a case.

Four days later, January 20th, I was again called to see the same patient and found her weak, prostrated, voiding black, inky liquid stools; tonsils much more swollen, the redness much darker about the arches of the palate, and a very few white specks on the tonsils and uvula. Gave the usual iron treatment for diphtheria, but yet believed that the case was a severe tonsillitis.

January 21st. Bowels constipated, throat entirely clear of all exudation.

January 22d. Some patches reappeared on the tonsils, red rings about the nostrils and dark red streaks underneath both eyes. Pulse, 120; temperature, 103½°, and slight milky appearance of mucous membrane of nostrils. Began inhalations of mild chloride of mercury.

January 23d. Throat entirely clear of all patches. Nostrils clean. Red rings about the nostrils beginning to disappear. Dark red lines underneath eyes also beginning to disappear. Very copious catarrhal discharge falling from posterior pharynx. An extensive bronchitis throughout both lungs, finger nails growing dark, skin assuming an ashy shade. Began giving stimulants—carb. amm., nux vomica, digitalis and spiritus frumenti.

January 24th. Patches in throat reappeared in abundance; all symptoms aggravated.

January 25th. More patches appeared, forming a continuous exudate. Cyanosis, or bluish condition of skin of entire body, being of a coffee color; nails very dark. Respiration, 40, short and labored; pulse, 130; temperature, 102°. Right lung almost completely infiltrated with exudate; respiratory murmur nearly absent; left lung almost as bad.

Dr. George E. Fell was called in consultation at 3 P. M., and agreed with me that he had never known a case to recover with similar symptoms in diphtheria. I had decided to use antitoxin, and after obtaining the consent of the family called in Dr. Fell, who had a supply which he had procured from the Pasteur Institute in New York City. This antitoxin was entirely prepared in this country, under the direction of Dr. Paul Gibier. Previous to the injection we both agreed the case was so desperate, that in event of failure it would be unjust to criticise the antitoxin treatment.

At 3 P. M. Dr. Fell injected four ordinary hypodermic syringefuls of the remedy, using an ordinary hypodermic needle without any difficulty. The injections were made into each shoulder and hip. The capacity of the syringe was three cubic centimeters, thus making twelve cubic centimeters for the first injection. The pulse at this time was 107; temperature, $101\frac{3}{4}°$; respiration, 42. At 7.30 P. M., same day, repeated the hypodermic medication, using fifteen cubic centimeters of the antitoxin. A slight change for the better was noted.

January 26th, at 4 A. M., I was notified that the child was dying, it having been changed from one bed to another; the slight exertion produced complete collapse, leading the family to believe the end had come. I directed a continuance of the supporting treatment, the same as ever, so long as the child could swallow. Soon afterward I telephoned Dr. Fell that the child would probably die and that it would not be necessary to make another visit until further notice. 10 A. M. I telephoned Dr. Fell that the child was better, and invited him to call and give the third treatment of antitoxin. This was done at 11 A. M. of the 26th inst. Marked improvement was noted in the patient, the temperature had fallen two degrees to 99¾° ; pulse not changed ; general appearance much better, movements of eyes more natural, child more interested in her surroundings, exudation on tongue and throat quite extensive but loose in character; lungs clearing up, respiratory area increased and expectoration quite free.

January 27th. The improvement still continued. At 11 A. M. temperature, 100½° ; pulse, 100. Cyanosis entirely passed away ; lungs almost entirely cleared up ; patient stronger, had taken some food ; heart's action much improved and at this time, about forty-eight hours after the antitoxin treatment had been instituted, a case hopeless in character, malignant, had been transformed into one which gave every hope of ultimate recovery.

January 27th. Evening, pulse, 97 ; temperature, 99½°.

January 28th. Afternoon, temperature normal, pulse, 97 ; lungs virtually cleared up.

This case should undoubtedly influence many in favor of the treatment who are now opposed to it, or do not believe in its efficacy ; it is not a renewal of the tuberculine or the Brown-Sequard craze. After an experience of twenty years, in which I have seen several hundred cases of diphtheria each year, this is the first time that I have ever known of a recovery from similar conditions. Dr. Fell, who has also had many cases of a zymotic nature, believed that the child would certainly have died without the antitoxin treatment.

It must be noted that the best treatment for diphtheria known to the profession was kept up from the beginning to the end of the treatment. The recovery, however, undoubtedly was due to the antitoxin. In this case a general desquamation of skin occurred during the patient's convalescence, a phenomenon quite unusual in diphtheria.

---

Salicylic Acid an Oxytocic.—Vineberg (*N. Y. Med. Jour.*) finds this special action of the drug upon the uterus to be sufficient to establish the following conclusions : Salicylic acid and its compounds may be found useful in scanty and delayed menstruation. They should not be administered to pregnant women who have a predisposition to abort or who suffer from menorrhagia and metorrhagia. Their administration should be watched carefully in all cases of pregnancy, and on the appearance of any "show," or anything resembling labor pains, they should be discontinued.--*Polyclinic.*

